# Supplementary material for: Decision Support Tools for Coronary Artery Calcium Scoring in the Primary Prevention of Cardiovascular Disease Do Not Meet Health Literacy Needs: A Systematic Environmental Scan and Evaluation
Source: Int J Environ Res Public Health. 2022 Sep 16;19(18):11705. doi: 10.3390/ijerph191811705 (PMC9517328; doi:10.3390/ijerph191811705)
Supplement: Supplementary file 1 [file ijerph-19-11705-s001.zip › ijerph-1845830-supplementary.pdf]

Supplementary Material

Table S1: List of known decision aid repositories and heart health/cardiology organization websites

**Organisation**	**URL**
Heart Foundation Australia	https://www.heartfoundation.org.au/
World Heart Foundation	https://world-heart-federation.org/
British Heart Foundation	https://www.bhf.org.uk/
Irish Heart Foundation	https://irishheart.ie/
Heart and Stroke Foundation Canada	https://www.heartandstroke.ca/
Singapore Heart Foundation	https://www.myheart.org.sg/
Heart Foundation NZ	https://www.heartfoundation.org.nz/
Heart Foundation South Africa	https://www.heartfoundation.co.za/
American Heart Association	https://www.heart.org/
Cardiac Society of Australia and NZ	https://www.csanz.edu.au/
Cardiac Health Foundation of Canada	http://www.cardiachealth.ca/
American College of Cardiology	https://www.acc.org/
British Cardiovascular Society	https://www.britishcardiovascularsociety.org/
Irish Cardiac Society	https://irishcardiacsociety.ie/
European Society of Cardiology	https://www.escardio.org/
Singapore Cardiac Society	https://www.singaporecardiac.org/
Canadian Cardiovascular Society	https://ccs.ca/
South African Heart Association	https://www.saheart.org/
Pan-African Society of Cardiology	https://www.pascar.org/
**Known Decision Aid repositories**	
Option Grid	https://www.optiongrid.org/
Decision Aid Library Inventory – Ottawa Research Institute	https://decisionaid.ohri.ca/
The Decision Box – Laval University	https://www.boitedecision.ulaval.ca/en/
NICE Decision Aids	https://www.evidence.nhs.uk/search?om=%5b%7b%22ety%22:%5b%22Patient%20Decision%20Aids%22%5d%7d,%7b%22srn%22:%5b%22National%20Institute%20for%20Health%20and%20Care%20Excellence%20-%20NICE%22%5d%7d%5d
NHS	https://www.nhs.uk/
Agency for Healthcare Research and Quality	https://www.ahrq.gov/

Additional sites identified during search: Cardiac Matters (www.cardiacmatters.co.uk), CardioSmart – American College of Cardiology (www.cardiosmart.org)

Table S2: Data extraction form

ID	
Title	
Developer	
Location	
Date of publication / latest update	
URL	
Format (e.g. interactive, static webpage, audiovisual)	
IPDASi criteria #1: Does the website describe the condition related to the decision?	Y/N
IPDASi criteria #2: Does the website describe the decision that needs to be considered?	Y/N
IPDASi criteria #3: Does the website identify the target audience?	Y/N
IPDASi criteria #4: Does the website list the options?	Y/N
IPDASi criteria #5: Does the website have information about the positive features of the options (e.g. benefits, advantages?)	Y/N
IPDASi criteria #6: Does the website have information about the negative features of the option (e.g. harms, side effects, disadvantages)?	Y/N
IPDASi criteria #7: Does the website help patients clarify their values for outcomes of options by: a) asking people to think about which positive and negative features of the options matter most to them AND/OR b) describing each option to help patients imagine the physical, social, and/or psychological effect	Y/N
What benefits are mentioned?	
What harms are mentioned?	
Advertisement included?	
Benefits: estimates of effect	
Harms: estimates of effect	
Visualisation of estimate of effect	Y/N – If yes, describe

Table S3: List of included online resources

Title	Developer	Location	Date of publication	URL
Coronary Calcium Scan: Should I Have This Test?	Healthwise	U.S.	April 2021 (Current as of this date)	https://decisionaid.ohri.ca/AZsumm.php?ID=1329 https://www.healthwise.net/ohridecisionaid/Content/StdDocument.aspx?DOCHWID=av2072
Do you need a calcium scan?	Harvard Health Publishing	U.S.	Aug-19	https://www.health.harvard.edu/heart-health/do-you-need-a-calcium-scan
Your Cholesterol Level Does Not Matter	Capital Cardiology Associates; written by Dr Lance E Sullenberger	U.S.	Not shown	https://capitalcardiology.com/cholesterol-level-not-matter/
The Heart Test You May Need—but Likely Haven't Heard of	Johns Hopkins Medicine	U.S.	Not shown	https://www.hopkinsmedicine.org/health/wellness-and-prevention/the-heart-test-you-may-need-but-likely-havent-heard-of
Do You Have the #1 Silent Killer of Women??	Covey Club; written by Lori Miller Kase	U.S.	Not shown	https://www.coveyclub.com/blog_posts/coronary-artery-calcium-scan-should-you-get-one/
Calcium-Score Screening Heart Scan	Cleveland Clinic	U.S.	1/05/2021 (last reviewed)	https://my.clevelandclinic.org/health/diagnostics/16824-calcium-score-screening-heart-scan
Do I Need a Coronary Calcium Score?	Texas Heart Institute; written by Stephanie Coulter	U.S.	Not shown	https://www.texasheart.org/heart-health/womens-heart-health/straight-talk-newsletter/do-i-need-a-coronary-calcium-score/
Cardiac CT for Calcium Scoring	Radiology Info	U.S.	January 2020 (last reviewed)	https://www.radiologyinfo.org/en/info/ct_calscoring

Heart scan (coronary calcium scan)	Mayo clinic	U.S.	Not shown	https://www.mayoclinic.org/tests-procedures/heart-scan/about/pac-20384686
CT Scan Screening for Heart Disease	Cardiac Matters; written by Jo Johnson	U.S.	January 2013 (last updated)	http://www.cardiacmatters.co.uk/the-role-of-ct-scanning-in-screening-for-heart-disease.html
Coronary Artery Calcium (CAC) Scoring	Cardio Smart; American College of Cardiology	U.S.	November 2018 (last edited)	https://www.cardiosmart.org/topics/high-cholesterol/exams-and-tests/coronary-artery-calcium-scoring
Coronary Artery Calcium Scoring	National Heart Foundation of Australia	Australia	Not shown	https://www.heartfoundation.org.au/Heart-health-education/Medical-Tests-Coronary-Artery-Calcium-Score
Coronary Artery Calcium Scans	Very Well Health; written by Richard N Fogoros	U.S.	Jul-20	https://www.verywellhealth.com/coronary-artery-calcium-scans-1745753

Table S4: Evaluation of individual online resources

Title	IPDASi Qualification Criteria							PEMAT-P		Readability			
	#1	#2	#3	#4	#5	#6	#7	Understandability	Actionability	SMOG (Grade)	% complex language	Passive voice	Lexical density
Coronary Calcium Scan: Should I Have This Test?	Yes	Yes	Yes	Yes	Yes	Yes	Yes	94%	83%	10	19.7	4	4
Do you need a calcium scan?	Yes	Yes	Yes	No	Yes	Yes	No	65%	0%	12	20.6	8	4.2
Your Cholesterol Level Does Not Matter	Yes	No	No	No	No	No	No	69%	80%	15	28.6	14	4.9
The Heart Test You May Need—but Likely Haven't Heard of	No	No	No	No	No	No	No	64%	60%	14	25.5	7	5.4
Do You Have the #1 Silent Killer of Women??	Yes	Yes	Yes	No	No	No	No	21%	0%	14	22	22	4.3
Calcium-Score Screening Heart Scan	Yes	Yes	Yes	Yes	No	No	No	88%	100%	13	31.4	10	4.1
Do I Need a Coronary Calcium Score?	Yes	Yes	Yes	No	Yes	Yes	No	71%	17%	13	27.8	8	4.9
Cardiac CT for Calcium Scoring	Yes	No	Yes	No	Yes	Yes	No	76%	83%	13	24	22	3.9

Heart scan (coronary calcium scan)	Yes	No	No	No	Yes	Yes	No	81%	60%	11	19.9	18	3.5
CT Scan Screening for Heart Disease	Yes	Yes	No	No	Yes	No	No	43%	0%	13	24.7	10	3.5
Coronary Artery Calcium (CAC) Scoring	Yes	Yes	Yes	No	Yes	No	No	86%	50%	12	22.9	14	3.8
Coronary Artery Calcium Scoring	Yes	No	No	No	No	No	No	75%	60%	12	21.8	8	3.3
Coronary Artery Calcium Scans	Yes	Yes	Yes	Yes	Yes	Yes	No	50%	33%	15	28.9	13	4.3
